# Cardiac T2* measurements in patients with iron overload: a comparison of imaging parameters and analysis techniques

**DOI:** 10.1186/1532-429X-13-S1-P302

**Published:** 2011-02-02

**Authors:** Phalla Ou, Yansong Zhao, Sara El Fawal, Puja Banka, Andrew J Powell

**Affiliations:** 1 Children's Hospital Boston, Boston, MA, USA; 2 Philips Healthcare, Cleveland, OH, USA

## Introduction

In patients at risk for iron overload, measurement of myocardial T2* has emerged as an important non-invasive tool to detect preclinical evidence of toxic levels and titrate chelation therapy. Nevertheless, there exists some variation among practitioners in cardiac T2* calculation methods.

## Purpose

To examine the impact of different imaging parameters and data analysis techniques on the calculated cardiac R2* (1/T2*) in patients at risk for cardiac siderosis.

## Methods

The study group consisted of 36 patients with thalassemia syndromes who had undergone clinical MRI assessment of cardiac siderosis using a standardized protocol and who were selected to yield a broad range of cardiac R2* values. Cardiac R2* measurements were performed on a 1.5 Tesla scanner using a ECG-gated, segmented, multiecho gradient echo sequence obtained in a single breath-hold. R2* was calculated from the signal intensity versus echo time data in the ventricular septum on a single mid-ventricular short-axis slice.

## Results

**Figure 1:**
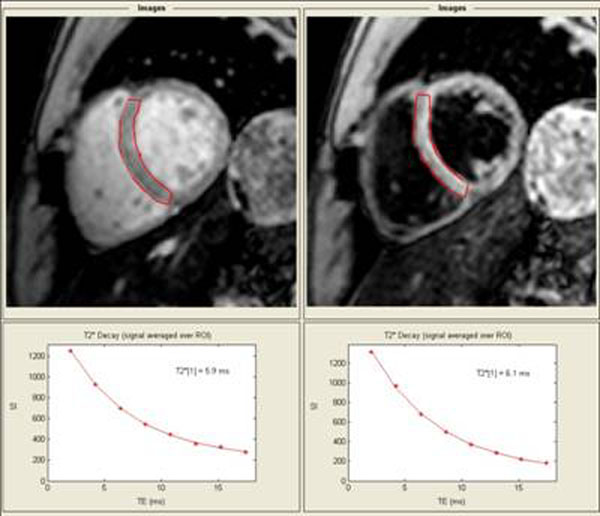
Typical short-axis mid-ventricular cardiac T2* images from the first echo (TE 2.0 ms) without and with a blood suppression pre-pulse in the same patient. Below, signal intensity versus TE is plotted for a region of interest encompassing the ventricular septum (outlined in red) along with the decay curve fit to a monoexponential with a constant offset model.

There was excellent agreement between R2* measured with a blood suppression pre-pulse (black blood technique) and without (mean difference 6.0±10.7 Hz). The black blood technique had superior within study reproducibility (R2* mean difference 1.6±8.6 Hz versus 2.7±14.6 Hz) and better interobserver agreement (R2* mean difference 3.4±8.2 Hz versus 8.3±16.5 Hz). Using the same minimum TE, the use of small (1.0 ms) versus large (2.2 ms) echo spacing had minimal impact on cardiac R2* (mean difference 0.3±8.7 Hz). The application of a region of interest versus a pixel-based data analysis had little effect on cardiac R2* calculation (mean difference 8.4±6.9 Hz). With black blood images, fitting the signal curve to a monoexponetial decay or to a monoexponential decay with a constant offset yielded similar R2* values (mean difference 3.4±8.1 Hz). Figure 1.

## Conclusions

The addition of a blood suppression pre-pulse for cardiac R2* measurement yields similar R2* values, and improves reproducibility and interoberver agreement. The findings regarding other variations may be helpful in establishing a broadly accepted imaging and analysis technique for cardiac R2* calculation.

